# Evaluation of the Influence of Formulation and Process Variables on Mechanical Properties of Oral Mucoadhesive Films Using Multivariate Data Analysis

**DOI:** 10.1155/2014/179568

**Published:** 2014-07-23

**Authors:** Hana Landová, David Vetchý, Jan Gajdziok, Petr Doležel, Jan Muselík, Kateřina Dvořáčková, Vladimír Jekl, Karel Hauptman, Zdeněk Knotek

**Affiliations:** ^1^Department of Pharmaceutics, Faculty of Pharmacy, University of Veterinary and Pharmaceutical Sciences Brno, Palackého třida 1/3, 612 42 Brno, Czech Republic; ^2^Avian and Exotic Animal Clinic, Faculty of Veterinary Medicine, University of Veterinary and Pharmaceutical Sciences Brno, Palackého třida 1/3, 612 42 Brno, Czech Republic

## Abstract

Oral mucosa is an attractive region for the local and systemic application of many drugs. Oral mucoadhesive films are preferred for their prolonged time of residence, the improved bioavailability of the drug they contain, their painless application, their protection against lesions, and their nonirritating properties. This work was focused on preparation of nonmedicated carmellose-based films using both solvent casting and impregnation methods, respectively. Moreover, a modern approach to evaluation of mucoadhesive films applying analysis of texture and subsequent multivariate data analysis was used. In this experiment, puncture strength strongly correlated with tensile strength and could be used to obtain necessary information about the mechanical film characteristics in films prepared using both methods. Puncture work and tensile work were not correlated in films prepared using the solvent casting method, as increasing the amount of glycerol led to an increase in the puncture work in thinner films. All measured texture parameters in films prepared by impregnation were significantly smaller compared to films prepared by solvent casting. Moreover, a relationship between the amount of glycerol and film thickness was observed, and a greater recalculated tensile/puncture strength was needed for an increased thickness in films prepared by impregnation.

## 1. Introduction

The oral cavity and its mucosa represent an attractive region for local and systemic applications of many active pharmaceutical ingredients. Direct absorption of drugs through the oral mucosa into systemic circulation circumvents undesirable hepatic metabolism (first-pass effect) and leads to higher bioavailability and faster onset of the action [1]. The lower enzymatic activity in the oral cavity compared to further parts of gastrointestinal tract limits the possibility of drug degradation, thus presenting yet another advantage [2].

The wide range of innovative dosage forms based on mucoadhesive polymers [3] for application to oral mucosa could be an attractive alternative to conventional dosage forms (oral rinses, gels, and pastes) for a number of reasons, such as extended residence time at the application site leading to prolonged therapeutic effect, easy manipulation, painless application, and possibility of preparation removal in the case of adverse drug effect [4]. Mucoadhesive oral films (MOFs) are preferred over mucoadhesive tablets because they are thin and flexible, which reduces uncomfortable feelings during normal activities such as eating, drinking, and speaking. Moreover, they can play the role of lesion dressings, which could minimize further irritation and reduce pain at the site of application [5].

MOFs as an innovative rapidly developed dosage form, recently also listed in* European Pharmacopoeia*, can be formulated for the application of a wide range of drugs for local or systemic therapy. Investigated mucoadhesive delivery systems for local action include, for example, antiseptics (chlorhexidine, cetylpyridinium chloride), antibiotics (ciprofloxacin, ofloxacin, and tetracycline), antifungal drugs (miconazole, nystatin, and clotrimazole), local anaesthetics (lidocaine, tetracaine), and other drugs for treatment of oral diseases such as recurrent aphthous stomatitis and others [6, 7]. Numerous drugs were investigated as possible active ingredients for oral mucoadhesive films with systemic action. These include peptidic hormones degradable in lower parts of the gastrointestinal tract (insulin, calcitonin, and oxytocin), antihypertensive drugs (metoprolol, carvedilol, nifedipine, and losartan), analgetics (particularly opioid drugs as buprenorphine or fentanyl—registered buccal films Onsolis and Breakyl), or drugs used for the treatment of asthma (salbutamol, terbutaline), diabetes (glipizide, glibenclamide), and other diseases [7].

The most widely used technology for the formulation of oral mucoadhesive films is the* solvent casting method* using homogenous dispersions of active ingredients and excipients. These liquid mixtures are poured into the casting moulds and the solvent is evaporated. Problems that may occur when employing this technology include inadequate rheological properties of the solution or suspension (high viscosity may affect dosing accuracy), entrapped air bubbles, insufficient content uniformity, and residual solvents presented in the final dosage form [2].

The second technology used for preparing MOFs is* hot-melt extrusion*, widely used in plastics and metal processing and the food industry. This method of MOF preparation was optimized by Repka et al. [8]. The principle of this method is controlled extrusion of the molten raw material through an orifice (the die) onto rolls which form the final homogenous sheets of film [9]. The described technology has many advantages in comparison with the solvent casting method, such as greater time effectiveness due to shorter processing time, no need of solvents (and therefore no solvent residues in the final dosage form), high stability of prepared films, and improved solubility and bioavailability of poorly soluble drugs. The relevant disadvantage is the requirement of thermal stability in all materials, moisture free components, limited and expensive excipients, and the requirement of specialized equipment [10]. Alternative methods for manufacturing MOFs consist of compressing or freeze-drying polymer powders and mixtures or printing active pharmaceutical ingredients onto a base film layer [11, 12].

A frequently discussed topic connected to MOFs is their evaluation, as authorized methods for testing this innovative medical form have still not been defined.* European Pharmacopoeia* describes only a dissolution test [13]. Authorised and justified methods for solid dosage forms (tablets, capsules, etc.) are usually unsuitable, cannot be used, or provide results which are irrelevant in the context of MOFs. Methods used to evaluate therapeutic transdermal systems seem to be suitable, but they must be significantly modified and special equipment is needed, for example, a modified physical balance for measuring mucoadhesive strength [14].

One suitable solution could be found in the innovative and sophisticated use of a texture analyzer, a special device widely used in the food and cosmetic industry, which has recently been also introduced into the pharmaceutical industry for evaluation of several dosage forms (tablets, semisolid preparations) and packaging (extrusion of ointment from the tube, etc.) [15, 16].

The texture (relations in the internal structure) of MOFs significantly affects their physicomechanical (strength, elasticity, durability, etc.) as well as mucoadhesive properties (residence time, water uptake, mucoadhesive strength, etc.), which together form the basis of dosage form with nonproblematic application, prolonged residence time, nonirritating properties, and easy handling and packaging, resulting in better patient compliance and higher therapy effectiveness.

For this reason, one of the modern trends in the evaluation of MOFs' properties could become the seeking of mutual relations between their texture and other* in vitro* or* in vivo* properties using multivariate data analysis.

There are two basic tests suitable for evaluation of films' durability against tension and compression using texture analyzer. The tensile strength represents the stress needed to stretch the film until it tears. For this assessment, a texture analyzer equipped with a special probe with two clamps is used. The influence of the cross-sectional area of the sample and the speed of upper clamp movement are recorded. The amount of the sample's deformation, in this case, namely, elongation, depends on the type and content of mucoadhesive polymer, the drug, and the amount of plasticizer [17]. Tear resistance and porosity, which also affect the mechanical resistance, depend upon the nature, type, and content of the polymer. It was observed that tear resistance increases with polymer concentration [18]. Using a texture analyzer with cylindrical probes, the puncture test can measure the resistance of the film against puncture. The area under the curve and the maximum stress or strength required to rupture the film determine its toughness [14].

The aim of the presented research was to analyze the texture of MOFs and then evaluate the influence of formulation and process variables on mechanical properties of prepared mucoadhesive films using multivariate data analysis. Until now, the number of known general dependencies related to tensile strength is limited. The presented research evaluates also the mechanical resistance of the films against puncture which is not usually published in scientific literature [19]. Moreover, the new modification of solvent casting method, the method of impregnation first described by Vetchý et al. [12], was used, evaluated, and compared to unmodified solvent casting method. The presented research illustrates the advantages of this innovative approach for evaluating oral mucoadhesive films' properties.

## 2. Material and Methods

### 2.1. Materials

Carmellose sodium (NaCMC), type Blanose 7LF Pharm, donated by Ashland Specialty Ingredients (Wilmington, USA) was used (in the form of water dispersion) as the basic mucoadhesive and film-forming polymer. Moreover for the method of impregnation, an acid form of carmellose in the form of nonwoven textile (Hcel HT) donated by Holzbecher Medical (Pardubice, CZ) was incorporated into the structure of the MOFs. Glycerol purchased from Dr. Kulich Pharma (Hradec Králové, CZ) was used as a plasticizer. All the other chemicals used in this experiment were of analytical grade.

### 2.2. Methods of Film Preparation

20 samples of mucoadhesive oral films with different concentrations of plasticizer (1–3%) were prepared using two different methods (Table 1). One-half of the samples were prepared by the standard solvent casting method and the second half using the innovative impregnation method. After drying, two different shapes of samples (25 × 25 mm and 10 × 40 mm) were punched from the prepared films for the testing of physicomechanical properties.

#### 2.2.1. Solvent Casting Method (Sample Series A and B)

Using an automatic pipette 18 mL (series A) or 27 mL (series B) in length, prepared uniform carmellose dispersions were cast into a round plastic mold (diameter 63 mm) and the solvent was left to evaporate at 30°C for 72 hours.

#### 2.2.2. Impregnation Method

Nonwoven carmellose textile was cut into circles 63 mm in diameter and placed into the casting molds. The textile was impregnated with an amount of prepared dispersion to ensure the weight of resulting films corresponded to films prepared using the solvent casting method (i.e., up to 18 mL in series C and up to 27 mL in series D). The solvent was similarly left to evaporate at 30°C for 72 hours.

### 2.3. Testing Methods

A modified method according to Shidhaye was used to evaluate the mechanical properties of the prepared films [20]. A Texture Analyzer CT3 (Brookfield, USA) equipped with a 4.5 kg load cell and TexturePro CT software was used to determine the tensile strength (ten. strength) of the prepared films. Rectangular samples (10 × 40 mm) were held between two clamps of probe TA-DGA positioned at a distance of 2 cm. The lower clamp was held stationary and the strips of MOF were stretched by the upper clamp moving at a rate of 0.5 mm/sec until the strip tore. The tensile work done during this process (ten. work) and the tensile deformation/elongation of the film at the moment of tearing (ten. def.) were also measured. The measurement was repeated three times for each film sample.

The texture analyzer with probe TA39 (a cylindrical probe with a diameter of 2 mm; probe motion speed 0.5 mm/sec) was used for the puncture test. The force needed to puncture square samples (25 × 25 mm) fixed in jig TA-CJ (puncture strength or pun. strength), the work done during this process (puncture work or pun. work), and the deformation of the film at the moment of puncture (puncture deformation or pun. def.) were measured. The measurement was repeated three times for each film sample.

Since the films were prepared in different ways (solvent casting method or impregnation method) and had different thicknesses, values measured by the texture analyzer were recalculated for a film thickness of 100 *μ*m for better comparison.

Film thickness was measured by microscopic analysis, using an optical microscope (STM-902 ZOOM, Opting, CZ) and NIS Elements software. A rectangular sample was vertically fixed in a holder, the microscope was focused on the edge of the film, and its thickness was measured at 5 places throughout the film. This was repeated 3 times for each film sample.

### 2.4. Methods of Data Analysis

The experiment was designed as a full factorial composed of 3 variables (glycerol; film thickness; nonwoven textile), where glycerol was used in 5 levels (1%; 1.5%; 2%; 2.5%; 3%), film thickness in 2 levels (18 mL, 27 mL), and nonwoven textile in 2 levels (presence—yes; absence—no). Data were initially checked using descriptive statistics. Subsequently, exploratory evaluation of data using PCA was carried out in order to study systematic variability in the data, relationships among variables and objects, and their correlations and to detect outliers. Prior to modeling, the variables were adjusted by autoscaling, that is, mean centering and scaling by standard deviation. MLR regression together with ANOVA was used to identify important variables and quantitative expression of their effect. Analysis was performed using the program Unscrambler X, v 1.3 (Camo software).

## 3. Results and Discussion

The results are summarized in Table 2.

The full data set of obtained results was analyzed using principal component analysis (PCA) models in order to describe the systematic variability. PCA is one of the oldest and most widely used methods to study dependencies in multivariate data set containing multiple variables and objects. Correlations of the original variables are evaluated on the basis of a smaller number of latent variables, the so-called principal component (PC), which represent a part of total variability. Their advantage is that they are independent (orthogonal), which greatly simplifies interpretation.

**Figure 1:**
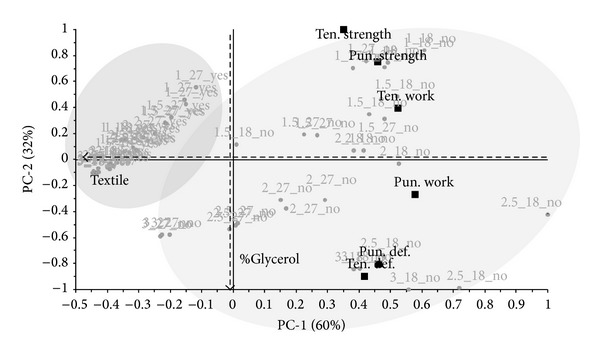
PCA biplot in Figure 1 shows the distribution of objects (circles) and variables (boxes) in the area of the first two components. Objects are labeled according to design parameters: %(glycerol)_ml(casted amount)_yes or no(non-woven textile). Groups with and without nonwoven textile are highlighted.

The PCA biplot (Figure 1) shows the variability of objects and variables in the area of the first two components. A presence/absence of nonwoven textile is shown by the formation of two groups along the components of PC-1. The effect of the amount of glycerol used is described along the components of PC-2. The first two components describe 92% of explained variance. With regard to the data structure, further PCA models were calculated separately for the data group with and without a nonwoven textile.

**Figure 2:**
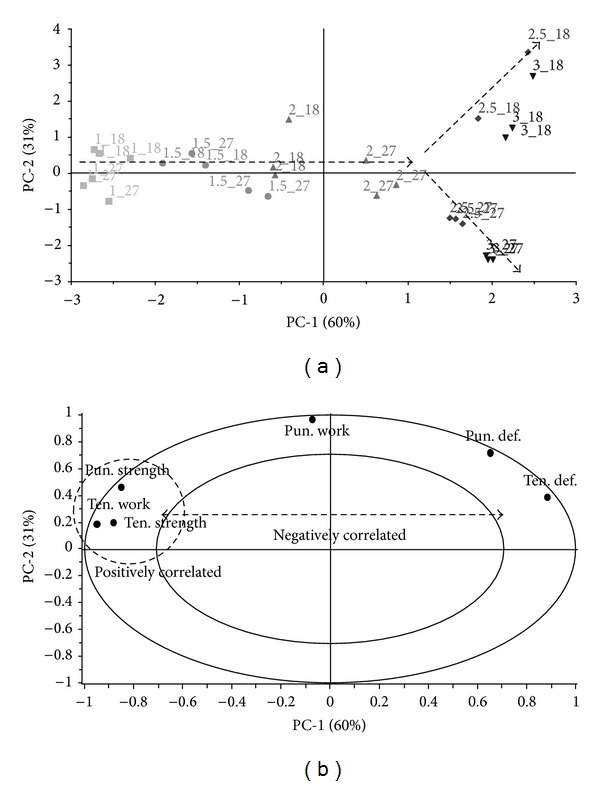
PCA; (a) scores plot; (b) correlation loadings plot, data of films without a nonwoven textile, positive correlation marked with a circle, and negative correlation indicated by an arrow. Objects are labeled according to design parameters: %(glycerol)_ml(casted amount).

A PCA model was constructed for data of films without a nonwoven textile. A systematic distribution of objects along the PC-1 depending on the content of glycerol in the films is shown in the scores plot (Figure 2(a)). The data distribution of the films with higher concentrations of glycerol (2.5%, 3%) is divided into two branches along the PC-2 component, which can be assigned to the variable pun. work. The correlation loadings plot (Figure 2(b)) together with the scores plot (Figure 2(a)) shows that puncture work was influenced by the thickness of the film differently compared to the rest of film characteristics, as the opposite effect in films containing 2.5–3% of glycerol was observed. The increased amount of glycerol in films C4 and C5 (18 mL) led to an increase in puncture work and conversely to a decrease in this work in films D4 and D5 (27 mL). The effect of the amount of glycerol used is well described by the parameters of the texture analysis located along PC-1 (Figure 2(b)). Moreover, some parameters are strongly correlated, both positively and negatively. Correlated parameters have the potential to explain a similar part of variability in the data. This may have the practical consequence of using only one of the parameters to obtain the necessary information about the film characteristics. Puncture strength, tensile strength, and tensile work positively correlated, and tensile deformation negatively correlated with these parameters.

**Figure 3:**
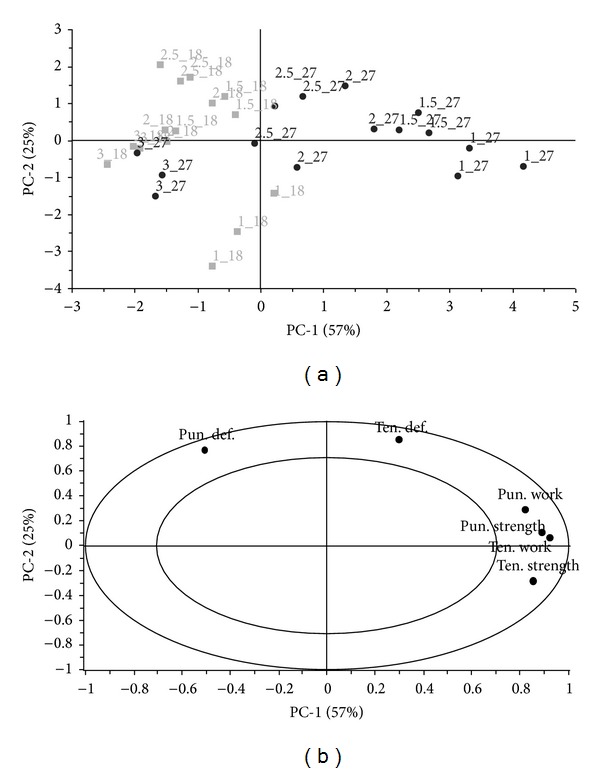
PCA; (a) scores plot; (b) correlation loadings plot, data of films with a nonwoven textile, positive correlation marked with a circle. Objects are labeled according to design parameters: %(glycerol)_ml(casted amount).

A separate PCA model for the data of films with a nonwoven textile was further calculated. The correlation loadings plot (Figure 3(b)) describes the relationship between variables in the data. In particular, variability of strongly correlated variables of tensile strength and tensile work, puncture strength and, in the specific case of these nonwoven textile films, puncture work are explained to some extent by the distribution of films into two groups according to their thicknesses (Figure 3(a)). Thicker films (27 mL) have a greater variability depending on the amount of glycerol as compared to thinner films (18 mL) which reflects different variance of each group along PC-1 and PC-2, respectively.

MLR regression was performed to better explain the effect of formulation parameters on the parameters measured by texture analysis. Using ANOVA (analysis of variance), model significance was tested and was also used to evaluate individual effects and interaction effects. Goodness of fit coefficients was used to evaluate the models: *R*-square described explained variance of the model, *R*-square of prediction expressed predictive ability of the model, and C.V. (coefficient of variation) was expressed as a percentage of the mean. MLR models were calculated for two levels of each variable (min, max), which made interpretation easier. The output of the selected MLR models was interaction plots which represented an average value effect of one factor dependent upon the level of the second factor.

**Figure 4:**
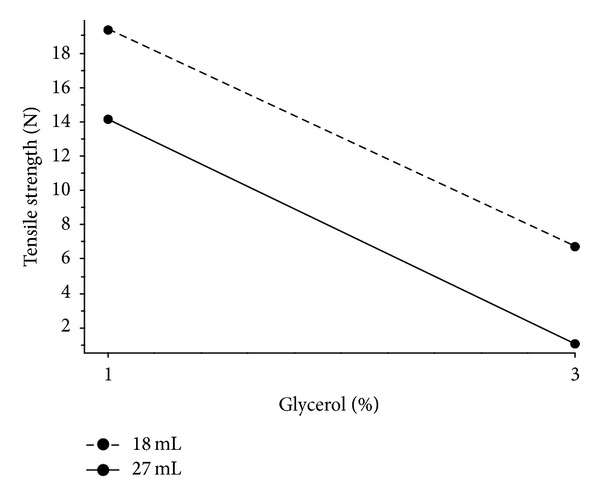
Interaction plot; effect of amount of glycerol on tensile strength at various thicknesses of films without nonwoven textile.

**Figure 5:**
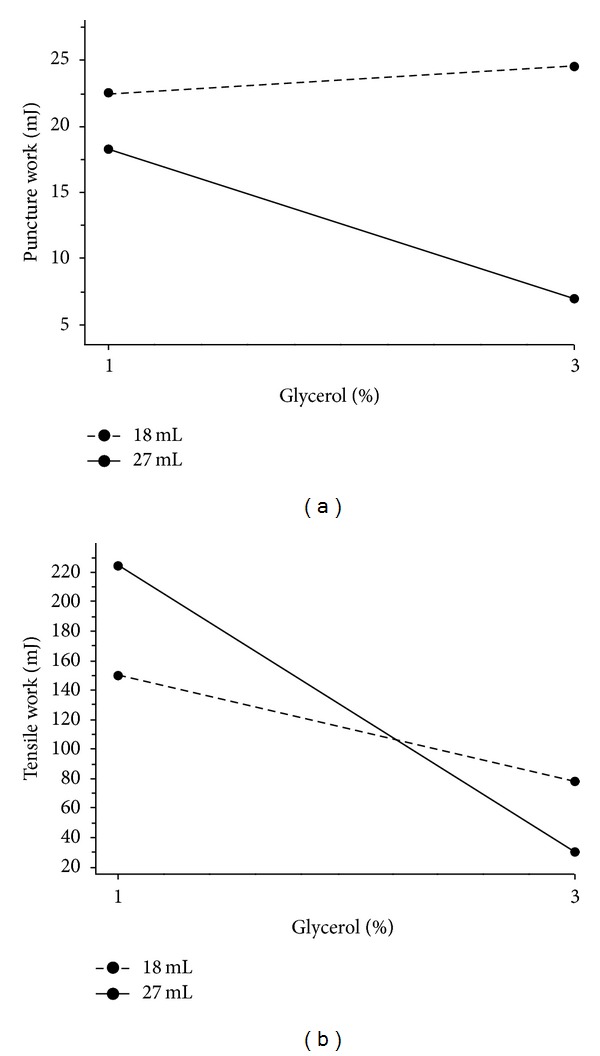
Interaction plot; (a) effect of the amount of glycerol on puncture work at various thicknesses of films without nonwoven textile; (b) effect of the amount of glycerol on tensile work at various thicknesses of films without nonwoven textile.

The characteristics of models for films without a nonwoven textile are summarized in Table 3. Until now, a general observation is known that the deformation/elongation of film increases with plasticizer content [14, 21]. In this experiment, it was found that puncture strength and tensile strength had similar regression characteristics. The effect of the interaction between the amount of glycerol and the film thickness did not occur as shown in Figure 4 where the lines are parallel; that is, increase in the amount of glycerol from 1% to 3% led to approximately the same decrease in the recalculated tensile strength for both film thicknesses. Greater recalculated tensile strength and puncture strength were needed for thinner films (dashed line). A greater recalculated tensile strength was also found for thinner films containing NaCMC and propylene glycol as plasticizer in Verma and Chattopadhyay's experiment [22]. Puncture work and tensile work were not correlated; therefore texture characteristics are manifested differently in graphs of interactions (Figures 5(a) and 5(b)).

**Figure 6:**
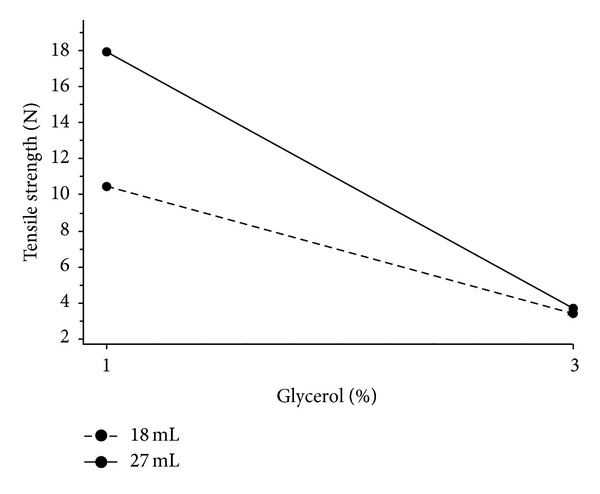
Interaction plot; effect of the amount of glycerol on tensile strength at various thicknesses of films with nonwoven textile.

**Figure 7:**
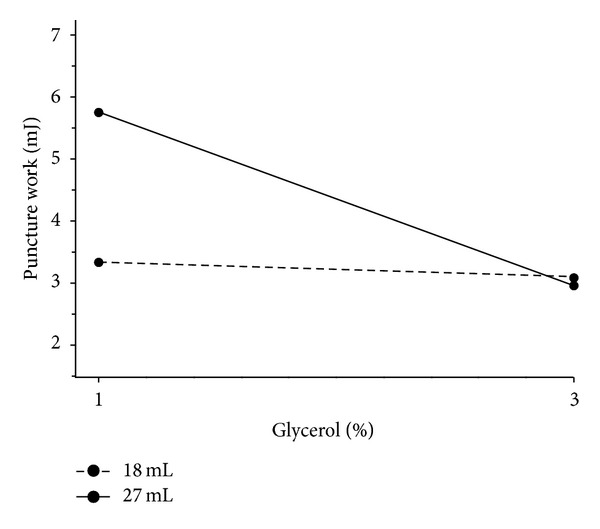
Interaction plot; effect of the amount of glycerol on the work done during film puncture at various thicknesses of films with nonwoven textile.

Characteristics of models for films with a nonwoven textile are summarized in Table 4. In general, all measured texture parameters were significantly smaller compared to films without the nonwoven textile. Unlike films without a nonwoven textile, interaction between the amount of glycerol and film thickness was observed. The results show that the best texture parameter for describing dependence between the amount of glycerol and film thickness was tensile strength with excellent regression characteristics, followed by puncture strength. These parameters were again strongly correlated but increasing the amount of glycerol from 1% to 3% did not lead to a decrease similar to that in the films without a nonwoven textile (Table 2).  Figure 6 shows that film thickness significantly affects tensile strength for 1% concentration of glycerol. Film thickness had practically no effect on films with 3% of glycerol. Greater recalculated tensile strength and puncture strength, respectively, were needed for thicker films (solid line), unlike films without nonwoven textile. The concentration of glycerol was a parameter which had a strong negative effect on tensile strength; that is, a high concentration of glycerol led to low tensile strength, regardless of the film thickness. Another suitable parameter to describe the dependence between the amount of glycerol and film thickness was tensile work. Again, film thickness affected tensile work and puncture work in films with a lower amount of glycerol and had almost no effect on films with higher amounts of glycerol (Figure 7).

## 4. Conclusion

New dependencies between formulation, process variables, and parameters describing mechanical properties of mucoadhesive films based on NaCMC were discovered. Puncture strength was strongly correlated with tensile strength and could be used to obtain necessary information about the mechanical characteristics of films. Puncture work and tensile work were not correlated in films prepared using the unmodified solvent casting method; increasing the amount of glycerol led to an increase in the puncture work in thinner films. All measured texture parameters in films prepared using the modified solvent casting method were significantly smaller compared to films without a nonwoven textile. Moreover, unlike films without a nonwoven textile, a relationship between the amount of glycerol and film thickness was observed, and greater recalculated tensile strength and puncture strength, respectively, were needed for thicker films with a nonwoven textile.

## Figures and Tables

**Table 1:** Formulations of mucoadhesive films.

Sample	NaCMC	Glycerol	Textile	Water	Casted amount
A1	2%	1%	No	97%	18 mL
A2	2%	1.5%	No	96.5%	18 mL
A3	2%	2%	No	96%	18 mL
A4	2%	2.5%	No	95.5%	18 mL
A5	2%	3%	No	95%	18 mL
B1	2%	1%	No	97%	27 mL
B2	2%	1.5%	No	96.5%	27 mL
B3	2%	2%	No	96%	27 mL
B4	2%	2.5%	No	95.5%	27 mL
B5	2%	3%	No	95%	27 mL
C1	2%	1%	Yes	97%	up to 18 mL∗
C2	2%	1.5%	Yes	96.5%	up to 18 mL∗
C3	2%	2%	Yes	96%	up to 18 mL∗
C4	2%	2.5%	Yes	95.5%	up to 18 mL∗
C5	2%	3%	Yes	95%	up to 18 mL∗
D1	2%	1%	Yes	97%	up to 27 mL∗
D2	2%	1.5%	Yes	96.5%	up to 27 mL∗
D3	2%	2%	Yes	96%	up to 27 mL∗
D4	2%	2.5%	Yes	95.5%	up to 27 mL∗
D5	2%	3%	Yes	95%	up to 27 mL∗

*The textile was impregnated with an amount of prepared dispersion to ensure the weight of resulting films corresponded to films prepared using the solvent casting method.

**Table 2:** Mechanical properties of mucoadhesive films.

Sample	Tensile testing			Puncture testing			Thickness (mm)
	Strength∗ (N)	Work∗ (mJ)	Deformation∗ (mm)	Strength∗ (N)	Work∗ (mJ)	Deformation∗ (mm)	
A1	26.55 ± 1.74	149.67 ± 31.31	9.18 ± 2.02	19.35 ± 0.86	22.55 ± 0.68	3.35 ± 0.07	83.76 ± 10.02
A2	18.54 ± 3.10	177.97 ± 29.62	14.27 ± 1.01	13.87 ± 1.08	20.20 ± 2.09	3.48 ± 0.11	101.69 ± 4.13
A3	14.07 ± 1.75	154.84 ± 24.40	19.77 ± 1.35	11.17 ± 2.31	23.29 ± 7.57	4.94 ± 0.71	109.90 ± 2.12
A4	7.43 ± 1.80	87.42 ± 11.11	35.56 ± 8.56	9.71 ± 2.89	34.64 ± 9.95	11.68 ± 0.36	83.84 ± 1.58
A5	4.06 ± 0.76	78.45 ± 22.73	35.78 ± 3.41	6.74 ± 0.95	24.53 ± 2.19	9.91 ± 0.34	112.05 ± 5.87

B1	20.77 ± 1.41	226.06 ± 11.19	6.50 ± 0.47	14.15 ± 1.79	18.33 ± 2.56	2.18 ± 0.20	145.70 ± 6.56
B2	15.86 ± 2.16	148.26 ± 42.46	12.04 ± 0.73	9.95 ± 1.38	21.39 ± 4.40	3.50 ± 0.22	156.18 ± 13.37
B3	5.94 ± 0.60	110.21 ± 11.47	21.02 ± 1.27	6.54 ± 1.36	21.57 ± 4.22	4.42 ± 0.07	205.13 ± 17.12
B4	5.83 ± 0.59	66.09 ± 1.83	24.71 ± 0.00	2.38 ± 0.30	12.05 ± 1.00	4.56 ± 0.17	250.85 ± 26.00
B5	0.82 ± 0.11	30.49 ± 4.56	20.89 ± 0.01	1.06 ± 0.11	6.97 ± 0.33	3.42 ± 0.14	296.64 ± 24.03

C1	10.45 ± 0.53	19.30 ± 5.16	0.96 ± 0.14	4.28 ± 0.45	3.35 ± 0.92	0.65 ± 0.08	268.17 ± 46.09
C2	7.69 ± 0.72	17.07 ± 3.32	1.22 ± 0.15	3.86 ± 0.52	4.28 ± 0.56	1.06 ± 0.03	221.46 ± 27.28
C3	5.29 ± 0.23	19.24 ± 3.85	1.18 ± 0.06	3.00 ± 0.12	3.75 ± 0.48	1.04 ± 0.04	246.93 ± 36.82
C4	4.59 ± 0.28	16.92 ± 4.28	1.41 ± 0.04	2.84 ± 0.41	3.93 ± 0.41	1.12 ± 0.07	217.33 ± 28.71
C5	3.39 ± 0.17	14.99 ± 1.69	1.10 ± 0.03	1.87 ± 0.25	3.09 ± 0.42	0.96 ± 0.04	257.23 ± 26.41

D1	17.96 ± 0.40	34.05 ± 2.33	1.22 ± 0.08	7.98 ± 0.59	5.74 ± 0.84	0.71 ± 0.05	220.00 ± 21.09
D2	12.07 ± 0.12	33.07 ± 2.86	1.31 ± 0.08	5.86 ± 0.57	5.68 ± 0.82	0.81 ± 0.06	266.97 ± 12.08
D3	7.65 ± 0.05	26.21 ± 6.85	1.30 ± 0.16	4.42 ± 0.56	4.82 ± 0.30	0.80 ± 0.07	302.97 ± 6.13
D4	4.36 ± 0.27	23.14 ± 4.04	1.25 ± 0.14	2.82 ± 0.55	4.20 ± 0.69	0.89 ± 0.09	315.90 ± 5.75
D5	3.71 ± 0.30	18.87 ± 4.92	1.13 ± 0.05	1.92 ± 0.12	2.97 ± 0.24	0.79 ± 0.11	322.70 ± 23.51

*Recalculated for 100 *μ*m film thickness.

**Table 3:** Models for films without nonwoven textile.

		Pen. strength	Pen. work	Pen. def.	Ten. strength	Ten. work	Ten. def.
Significance (*P* value)	Model	0.000	0.000	0.000	0.000	0.000	0.000
	mL of glycerol	0.000	0.002	0.000	0.000	0.000	0.000
	Thickness	0.000	0.000	0.000	0.000	0.284	0.000
	mL of glycerol ∗ thickness	0.711	0.000	0.000	0.100	0.001	0.009

Goodness of fit	*R*-square	0.98	0.96	0.97	0.99	0.95	0.97
	*R*-square prediction	0.96	0.91	0.93	0.98	0.89	0.93
	C.V. in %	10.65	9.55	14.22	9.05	16.53	14.79

**Table 4:** Models for films with nonwoven textile.

		Pen. strength	Pen. work	Pen. def.	Ten. strength	Ten. work	Ten. def.
Significance (*P* value)	Model	0.000	0.003	0.006	0.000	0.000	0.049
	mL of glycerol	0.000	0.004	0.002	0.000	0.000	0.803
	Thickness	0.000	0.018	0.284	0.000	0.002	0.029
	mL of glycerol ∗ thickness	0.000	0.012	0.033	0.000	0.007	0.052

Goodness of fit	*R*-square	0.98	0.81	0.77	1.00	0.90	0.61
	*R*-square prediction	0.96	0.58	0.49	0.99	0.77	0.11
	C.V. in %	9.83	17.64	9.97	4.67	14.46	8.15
